# RNA Order Regulates Its Interactions with Zwitterionic Lipid Bilayers

**DOI:** 10.1021/acs.nanolett.4c04153

**Published:** 2024-12-24

**Authors:** Akhil Pratap Singh, Janak Prabhu, Stefano Vanni

**Affiliations:** † Department of Biology, University of Fribourg, Chemin du Musée 10, CH-1700 Fribourg, Switzerland; ‡ Swiss National Center for Competence in Research (NCCR) Bio-inspired Materials, University of Fribourg, Chemin des Verdiers 4, CH-1700 Fribourg, Switzerland

**Keywords:** RNA, lipid bilayers, H-bonding, entropy, lipid−oligonucleotide conjugates

## Abstract

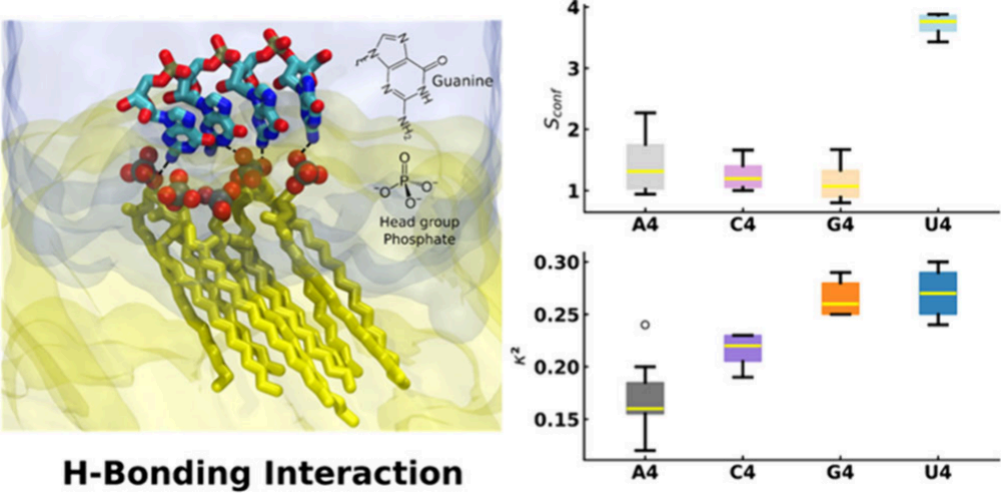

RNA–lipid interactions directly influence RNA activity, which plays a crucial role in the development of new applications in medicine and biotechnology. However, while specific preferential behaviors between RNA and lipid bilayers have been identified experimentally, their molecular origin remains unexplored. Here we use molecular dynamics simulations to investigate the interaction between RNA and membranes composed of zwitterionic lipids at the atomistic level. Our data reproduce and rationalize previous experimental observations, including that short-chain RNAs rich in guanine have a higher affinity for gel-phase membranes compared to RNA sequences rich in other nucleotides and that RNA prefers gel-phase membranes to fluid bilayers. Our simulations reveal that RNA order is a key molecular determinant of RNA–zwitterionic phospholipid interactions. Our data provide a wealth of information at the atomic level that will help accelerate research on RNA–lipid assemblies for task-specific applications such as designing lipid-based nanocarriers for RNA delivery.

RNA oligomers play a crucial role in controlling several cellular functions, including cell death and proliferation,^1^ and they are associated with the onset of several types of cancer and other genetic disorders in cells.^2−5^ As a consequence, RNA-based therapeutics have experienced significant progress in the past few years, leading to a breakthrough in gene therapy approaches for the treatment of a wide range of diseases.

RNA requires robust delivery systems to prevent the loss of RNA activity,^6^ and phospholipid–RNA conjugates have shown great promise as delivery carriers.^4,7−10^ Yet, how lipids interact with various RNA-based therapeutics, such as mRNA or small interfering RNA (siRNA) at the molecular level, remains mostly unclear.

Experimental studies have demonstrated that nucleotide sequence and structure greatly influence molecular interactions in phospholipid–RNA conjugates.^11−19^ For example, in lipid nanoparticles, siRNA resides on the surface of conjugates and mRNA at its center.^11−13^ Specifically, such interactions are strongly influenced by electrostatics, since RNA has a negative charge on the phosphates (PO_4_^–^) and numerous cellular lipids have a variety of differently charged head groups,^18^ including lipids with negatively to zwitterionic charged moieties.^19^

In addition to electrostatic interactions, several reports have shown that RNA/DNA adsorption at the bilayer interface can also be controlled by acyl chain order,^20−28^ with randomized mixtures of RNA showing a preferential binding affinity for membranes in the solid crystalline (gel) phase.^20,26^ Moreover, recent studies have revealed that specific RNA sequences (guanine rich) display higher binding affinity for gel membranes in the presence of low salt concentrations.^22^ Additionally, it has been reported that RNA–lipid binding regulates RNA activity^29,30^ and that lipids can provide RNA-safeguarded and selective microcompartments^31,32^ through membranous structures due to their amphiphilic nature; specifically, RNA could reside on membrane surfaces as a consequence of direct RNA–lipid interactions, providing a specialized physicochemical microenvironment to maintain RNA activity.^30^

However, none of the studies described above, presumably due to the inherent limitations involved in the techniques used, provided a detailed explanation of the molecular origin of these specific RNA–zwitterionic lipid interactions. To investigate this aspect, here, we opted to use all-atom (AA) molecular dynamics (MD) simulations, as this computational technique can provide an atomic-level description of complex chemical systems under highly controlled conditions, complementary to experimental methods. By focusing on the detailed understanding of the RNA–lipid binding as a function of the primary sequence, structure, and length of RNA as well as on the nature of the membrane (ordered vs liquid phase), we found that RNA order is the key property modulating binding of RNA to zwitterionic phospholipid bilayers. Our study provides useful information at the molecular to atomic level, which could be helpful to design robust lipid nanoparticles as efficient drug delivery systems.

## Guanine-Rich Oligomers Exhibit Greater Adsorption at DPPC-Gel Phase Bilayers

**Figure 1:**
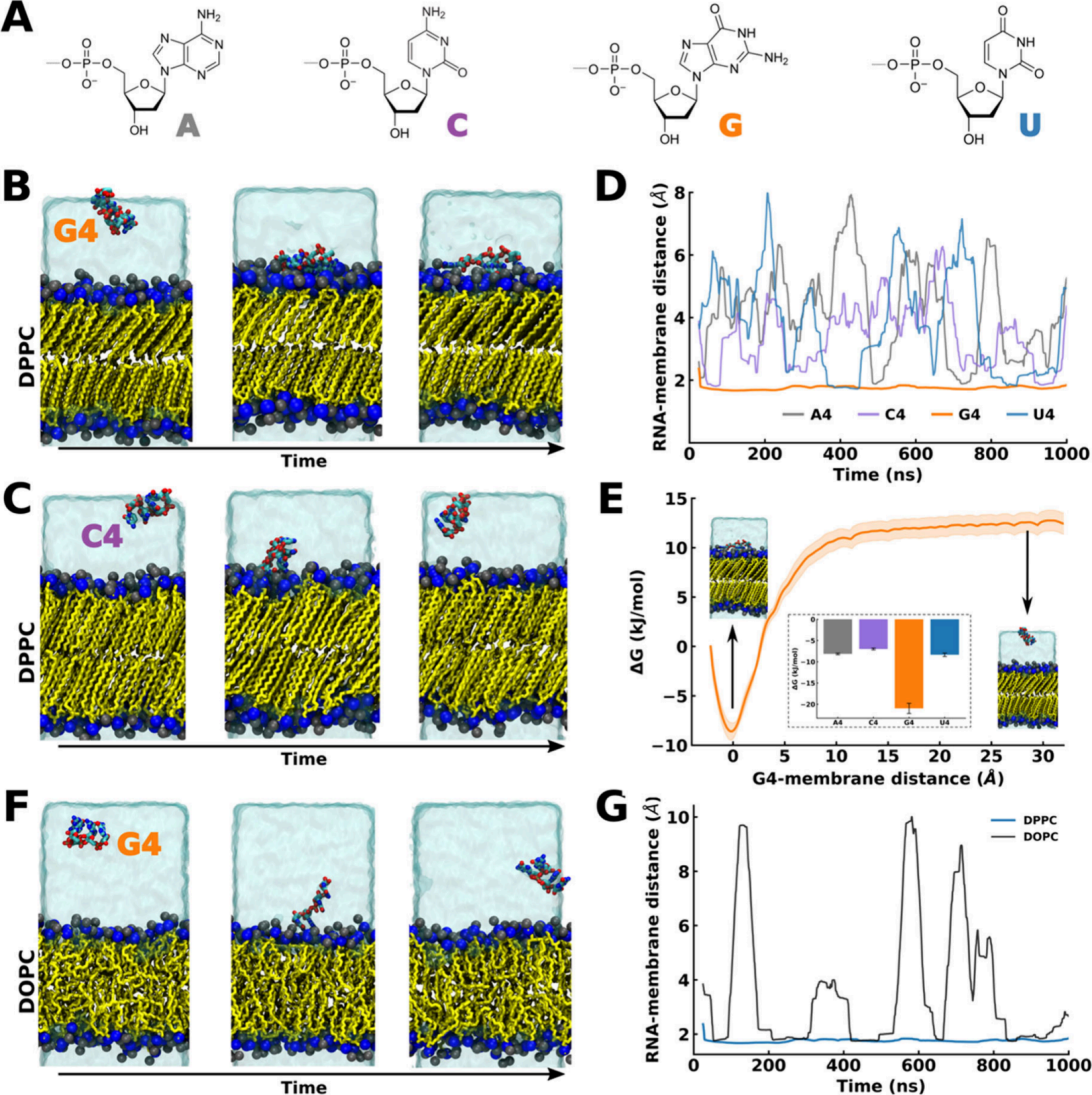
G4, but not A4, C4, and U4, stably binds to DPPC-gel lipid bilayers. (A) Molecular structures of RNA nucleobases: adenine, cytosine, guanine, and uracil. (B, C) Representative snapshots of the time evolution of MD simulation of (B) G4 oligomer/DPPC-gel bilayer and (C) C4 oligomer/DPPC-gel bilayer systems. Color scheme: Blue and black are for nitrogen and phosphate atoms of lipid head groups, yellow is for lipid tails, and translucent ice blue is for water. Oligomers (G4 or C4) are in multicolored licorice representation. Counter ions are removed for clarity. (D) Running average of the minimum distance between oligomers and the DPPC-gel bilayer over simulation time. (E) Potential of mean force (PMF) for G4 oligomer binding to the bilayer surface along the bilayer normal (*z*-axis), with snapshots of bound and unbound states from the umbrella sampling simulations. Inset: free energy difference between solution and membrane surface configuration for A4, C4, G4, and U4 oligomers from umbrella sampling simulations. (F) Representative snapshots of the time evolution of MD simulation of the G4/DOPC-liquid bilayer system. The color scheme is identical with that of (B). (G) Running average of the minimum distance between oligomers and the lipid bilayer in the G4/DPPC-gel bilayer and G4/DOPC-liquid systems.

To investigate the molecular origin of the interactions between RNA and lipid bilayers, including the puzzling specificity for guanine observed experimentally,^22^ we initially performed AA-MD simulations of RNA oligomers, with variations in their sequence and structure, on DPPC-gel phase systems. We simulated four different short-chain oligomers, with each system containing a homo-oligomer composed of a four-nucleotide sequence (4-nt): adenine (A4), cytosine (C4), guanine (G4), and uracil (U4) (Figure 1A). These simulated systems, their compositions, and number of replicates are summarized in Table S1, and method details can be found in the Supporting Information.

Analysis of the MD trajectories of all our replicas reveal that guanine-rich oligomers (G4) easily adsorb on bilayer interfaces (Figures 1B,D; S1, S2, and S3). G4 adsorption is maintained during the time scale of our simulations (Figure 1D). In contrast, the interaction of C4, A4, and U4 oligomers with the interface is short-lived or absent (Figures 1C, D; S1, S2, and S3), as these oligomers do not create stabilizing interactions at the interface, quickly returning to the aqueous phase (Figure 1C,D).

To characterize the preference of the different oligomers for the DPPC-gel bilayer, we first computed the interaction energy (*E*_Coul._ + *E*_LJ_) between the different oligomers and the DPPC-gel bilayer over time (Figure S2C). This analysis indicates that the interaction between G4 and the bilayer has a strong enthalpic component, achieved via the establishment of electrostatic and Lennard-Jones interactions between the oligomer and the lipid head groups. Next, to explicitly quantify the binding free energy of all oligomers to the DPPC-gel bilayer (Figure 1E), we performed umbrella sampling calculations using the distance between the center of mass (COM) of oligomers and the bilayer as the reaction coordinate. This analysis also identifies that G4 has a clear preference for the bilayer interface compared to all other oligomers (approximately 22 kJ/mol vs 8 kJ/mol; Figure 1E).

Overall, all these analyses indicate that G4 mainly localizes at the water–bilayer interface, while all other oligomers (C4, A4, U4) interact with the bilayer only very transiently (Figures 1, S1, and S2). Of note, control simulations with physiological ion concentrations and in the presence of divalent ions (150 mM NaCl + 50 mM MgCl_2_) indicate that these results are qualitatively similar in the presence of physiological ionic conditions, with G4 stably binding DPPC-gel bilayers more readily than A4. However, a moderate binding of A4 to the DPPC-gel bilayer at this ionic strength, which is significantly higher than that used experimentally,^22^ could be observed (Figure S3).

Finally, we investigated the interaction of G4 with fluid DOPC bilayers, as short nucleotide oligomers were shown experimentally to strongly prefer gel-phase vs fluid-phase bilayers^22^ (Figure 1F,G). In agreement with experimental observations, we indeed observed significantly lower membrane binding to DOPC bilayers for G4, as indicated by the time trace of the minimum distances between G4 and the bilayer along the MD trajectory and by the G4-bilayer distance probability distribution with time (Figure 1G and Figures S4, S5). Overall, our data indicate that nucleotide oligomers preferentially interact with DPPC-gel bilayers rather than with fluid DOPC bilayers and that G4 establishes preferential interactions with DPPC-gel bilayers compared to all other nucleobases, in agreement with experimental measurements.^22^

## Base-Pairing Promotes Binding of RNA to Gel-Phase Zwitterionic Lipid Bilayers

**Figure 2:**
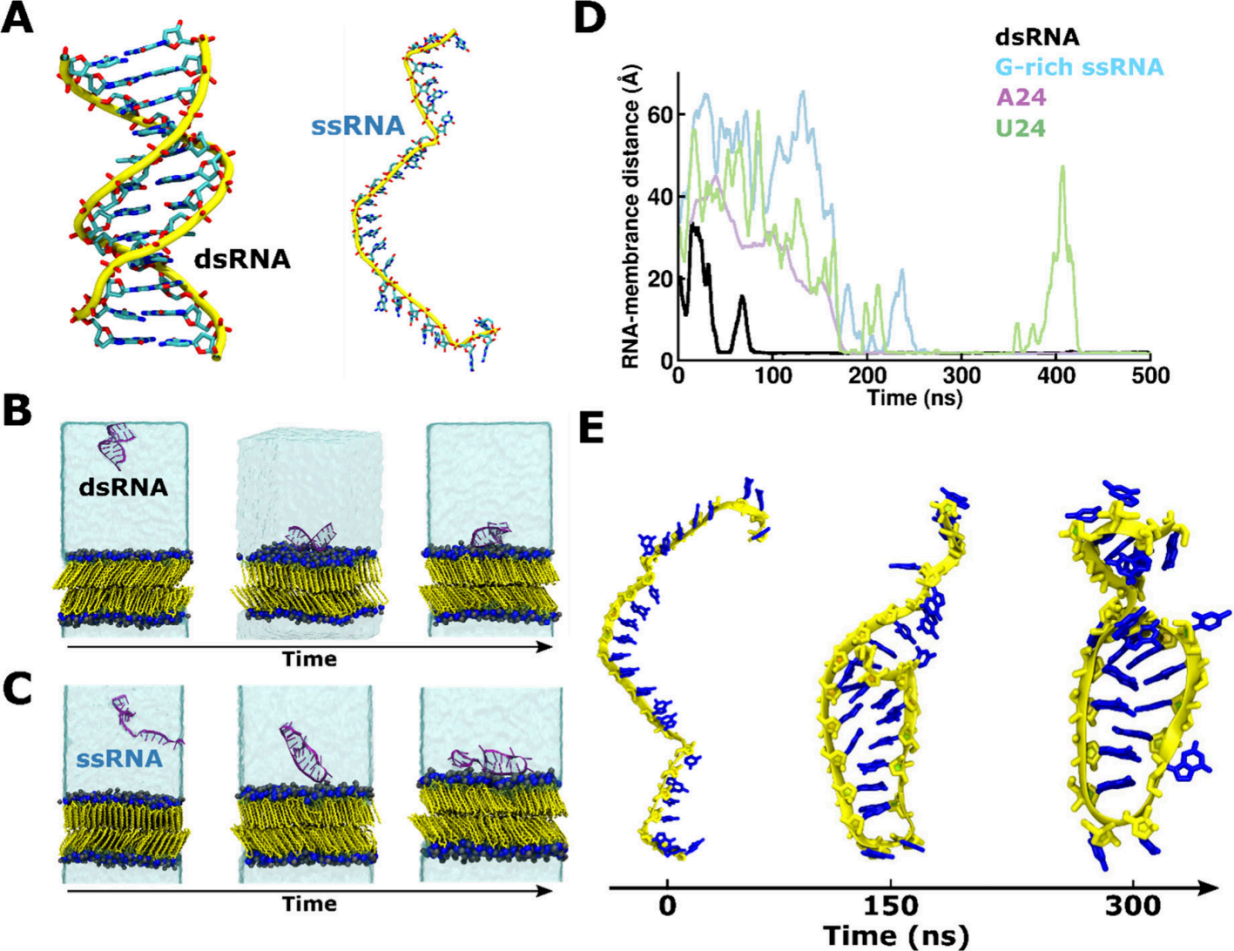
dsRNA and ssRNA adsorption on DPPC-gel membranes. (A) Structure of simulated (i) dsRNA and (ii) ssRNA. (B, C) Time evolution of MD simulations of (B) dsRNA/DPPC-gel and (C) G-rich ssRNA/DPPC-gel systems. Color scheme: blue and black for nitrogen and phosphate atoms of lipid head groups, yellow for lipid tails, translucent ice blue for water. RNA structures are depicted in a new-cartoon (magenta) representation. (D) Time evolution of the minimum distance between dsRNA or ssRNA and the DPPC-gel bilayer in MD simulations. (E) Structural arrangement of G-rich ssRNA in the ssRNA/DPPC-gel phase bilayer system over time, at (i) *t* = 0 ns, (ii) *t* = 150 ns, and (iii) *t* = 300 ns. Color scheme: yellow for ssRNA backbone and blue for nucleic bases.

We next investigated larger-scale RNA structural effects on RNA-bilayer binding and specifically the decreased propensity of single-stranded RNA (ssRNA) vs double-stranded RNA (dsRNA) to bind to DPPC-gel bilayers observed experimentally.^22^ To this end, we simulated three 24-nt ssRNAs—one cytosine/guanine (GC)-rich, one uracil, and one adenine homo-oligomers—and one dsRNA (Figure 2A) with a DPPC-gel bilayer. We took three different ssRNA, as they exhibit sequence-dependent ordered and disordered conformations.^33,34^ Specifically, both the adenine homo-oligomer and the cytosine/guanine (GC)-rich ssRNA are expected to form ordered structures due to favorable base-pairing or stacking interactions, while the uracil homo-oligomer is expected to remain unfolded.^33^ For dsRNA, we simulated multiple dsRNA structures obtained from the PDB in an aqueous environment to confirm their duplex stability over time. After proper screening, dsRNA from PDB ID 4JRT was chosen as a model for these simulations. System details are given in Table S1, while Table S2 lists the RNA sequences used.

Analysis of the MD trajectories of both dsRNA/DPPC and ssRNA/DPPC systems indicates that all systems adsorb on the membrane interface (Figure 2B, C, and D). This observation is consistent with experimental observations,^22^ as the RNA/lipid ratio in our *in silico* systems (12 lipids per nucleotide) is above the threshold at which membrane binding is experimentally observed for both dsRNA and ssRNA.^22^ However, analysis of the time traces indicates that dsRNA always binds to the DPPC-gel bilayer within a few tens of nanoseconds of simulation, staying there for the rest of the simulation (Figures 2B,D; S6), while binding of ssRNA is delayed compared to that of dsRNA (Figure 2C,D).

Further, analysis of the trajectories reveals that in all our replicas, G-rich and adenine-homo-oligomers (Figure S7A,B and Figure S8A,B) switch to a stable, ordered structure (comparable to that of a hairpin) in a sequence-dependent manner,^33,34^ somewhat analogous to dsDNA, before adsorption on the bilayer (Figure 2C,E and Figure S9A,B). On the contrary, U24-ssRNA, in agreement with experimental observations,^33^ displays a persistent disordered structure and long-delayed adsorption in all replicas (Figure S10A,B,C).

To investigate the molecular origin of the different dsRNA and ssRNA-U24 adsorption mechanisms, we focused on the two major interactions driving RNA binding to lipid bilayers: (i) charge–charge interactions between the negatively charged phosphate groups on the oligomer backbone and the positively charged moieties of lipid headgroups and (ii) hydrogen bonds between the nucleotide side chains and the phospholipid head groups. We found that charge–charge interactions are the dominant energy term for the initial RNA–membrane adsorption (Figure S11A) and that while direct H-bonds between dsRNA and the lipid bilayer are almost absent throughout the trajectory, H-bond interactions between U24 and the DPPC-gel bilayer become an important contribution in the later stages of U24 adsorption (Figure S11C), resulting in an increase in enthalpic interaction with the bilayer (Figure S11B). Overall, our simulations suggest that the mechanism of RNA adsorption correlates with its secondary structure, suggesting that predefined ordered states might preferentially accelerate its binding to DPPC-gel lipid bilayers. As a preliminary confirmation of this hypothesis, we observed that ssRNA binds to bilayers much quicker in the presence of high salt concentrations and divalent cations, as these conditions promote a stabler structure for ssRNA oligomers (Figure S12).^33,35−41^

## RNA Order Facilitates the Interaction between RNA and Gel-Phase Lipid Bilayers

The observation that ssRNA sequences such as G-rich and A24 oligomers fold into ordered structures before binding to DPPC-gel bilayers suggests that the ssRNA disorder might antagonize its binding to lipid bilayers. To assess the generality of this observation, we next tested whether this holds true also for the simple case of the 4 small RNA oligomers (A4, C4, G4, and U4) we previously investigated and for which membrane binding was observed exclusively for the oligomer G4 (Figure 1).

**Figure 3:**
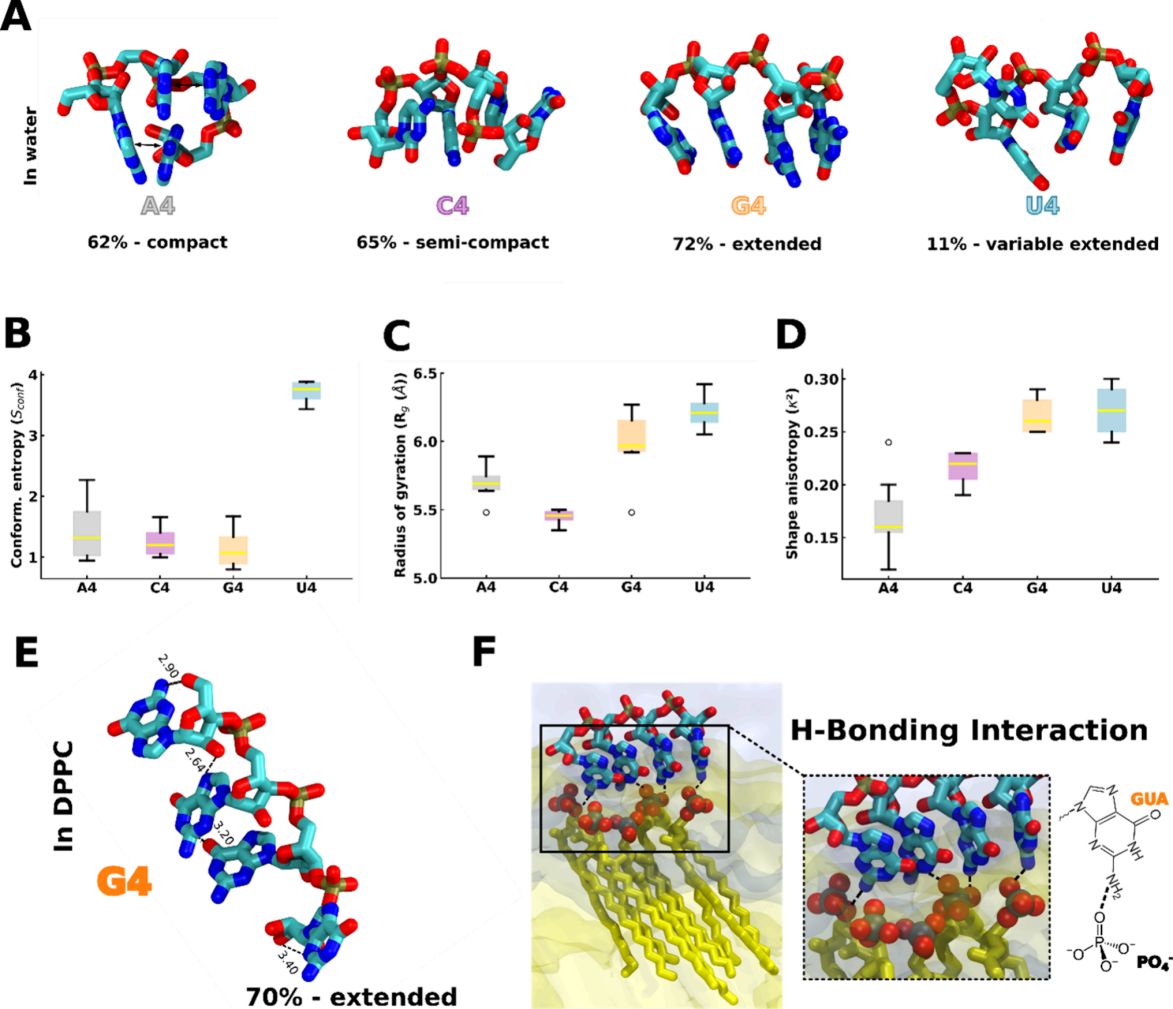
Conformational entropy of RNA oligomers. (A) Most populated conformations of the different oligomers from cluster analysis in bulk water phase from the last 600 ns of MD simulations (1000 structures each). (B) Oligomer conformational entropy determined from cluster analysis. Color scheme: gray for A4, purple for C4, orange for G4, and blue for U4 oligomers. (C) Radius of gyration calculated during the MD simulation for different oligomers in the bulk water phase. (D) Relative shape anisotropy κ^2^ for all oligomers in the bulk water phase. (E) Spatial arrangements of the G4 oligomers in the G4 oligomer/DPPC-gel system from MD simulations. Color scheme: red for oxygen, cyan for carbon, and blue for nitrogen. For clarity, the hydrogens of oligomers are omitted. The dotted lines represent the H-bonds. (F) G4 oligomer arrangement at the DPPC-gel bilayer interface from MD simulations. The representative MD snapshot of the G4/DPPC-gel bilayer shows the probable H-bonding interaction between the guanine–NH_2_ group and lipid phosphate oxygen atoms, at the interface. Color scheme: red for oxygen, blue for nitrogen, cyan for carbon, brown for phosphate, and yellow for lipid tails. Licorice representation (except lipid’s tails) is used for G4 oligomers. The nitrogen, oxygen, and phosphate atoms of lipid are represented by spheres. The translucent yellow and ice blue represent the DPPC bilayer and water phases. For clarity, only a few lipids of DPPC bilayers are shown here and ions are omitted.

To this end, we focused on the order/disorder behavior of the 4 small RNA oligomers (A4, C4, G4, and U4) in solution. To do so, we computed their conformational entropy by assessing their microstate populations using cluster analysis.^42^ This analysis indicates that while U4 is highly disordered, all other short oligomers (A4, C4, and G4) adopt one dominant population in solution and have low conformational entropies (Figure 3A,B). However, structural analysis of these conformations indicates that the G4 oligomer adopts a much more extended structure in solution, as indicated by its radius of gyration (Figure 3C) and its shape anisotropy (Figure 3D).

Notably, cluster analysis of the G4 oligomer when bound to the DPPC-gel bilayer (Figure 1) indicates that the same structure that is populated in solution is also the most abundant conformation in the G4-DPPC-gel system (Figure 3E, F, and A). This unique behavior of G4 is likely a consequence of its ability to form multiple H-bonds compared to other nucleotides (Figure S13). This allows G4 to establish multiple hydrogen bonds with the DPPC-gel bilayer (Figures 3F, S13) while also retaining multiple intramolecular H-bonds that keep it in an extended conformation (Figure 3F).

Interestingly, since the area per lipid of the DPPC-gel bilayer is much smaller compared to that of the DOPC bilayer (50 Å^2^ vs 68 Å^2^), thus enhancing local surface charge density by 25% in the case of the DPPC-gel bilayer, it appears that the ability of ssRNA to adopt a structure that matches the charge distribution of the bilayer is a key molecular requirement to promote its binding to the membrane.

In summary, here we used atomistic MD simulations to investigate the adsorption behavior of different RNA structures on zwitterionic lipid membranes and to determine the molecular details of the RNA–zwitterionic lipid interactions. While the role of negatively charged lipids and of RNA–lipid electrostatic interactions has been widely investigated in the past, our work emphasizes the binding mechanism of RNA toward gel and fluid-phase lipid bilayers that are devoid of negatively charged lipids. The rationale behind this choice is twofold: not only have these interactions been shown to be important experimentally,^22^ but they also could be leveraged to engineer RNA–lipid nanoparticles with targeted physicochemical properties and RNA activity.

In agreement with experimental observations,^22^ we observed that guanine-rich short oligomers have higher affinity for DPPC-gel phase membranes compared to other nucelobases. We found that guanine-rich oligomers prefer extended conformation in solution with low conformational entropy, a combination that is achieved thanks to the large number of H-bond acceptor and donor atoms in its molecular skeleton compared to those of other nucleobases. This facilitates the lipid–RNA interaction through more exposed interaction sites and via the matching of the periodicity of polar heads in gel bilayers.

We also observed that dsRNA adsorbs rapidly on DPPC-gel bilayers, likely as a consequence of the perfect matching between the exquisite periodicity of dsRNA that is achieved through base pairing and that of gel lipid bilayers. Unexpectedly, we also observed that the ability of ssRNA to fold into ordered structures promotes its adsorption on the lipid bilayer. Overall, our results are consistent with previous theoretical considerations suggesting that RNA behavior is modulated by the combination of electrostatic interactions, hydrogen bonding, and interactions with ions.^35−41,43−45^ However, our simulations also suggest that these interactions not only play a direct role in determining RNA adsorption to lipid bilayers but also indirectly modulate this process by strongly affecting RNA conformational plasticity and order/disorder behavior.

The main limitations of our MD simulations originate from the potential inaccuracy of the employed force field and from the imperfect matching of the experimental and simulation conditions, e.g., for what pertains to RNA/lipid ratio, salt type, and concentration as well as insufficient sampling. On the other hand, our simulations provide interesting insights beyond the specifics of RNA–lipid adsorption. First, the excellent agreement of our data with experimental observations on RNA–lipid bilayer interactions validates *a posteriori* the quality of our force fields and simulation parameters, paving the way for future studies of RNA–lipid interactions. Second, our observations raise interesting and practical ideas on how to optimize RNA–lipid interactions for delivery systems: (i) ssRNA binding could be accelerated by increasing the number of nucleobases in the sequence that can do canonical base pairing. Overall, base pairing plays an essential role in adsorption, as we observed in the dsRNA and structured ssRNA in this study. (ii) RNA membrane adsorption, and especially of disordered ssRNA, can be improved by increasing nucleotides that have H-bond donor and acceptor capability, such as guanine or, alternatively, adenine. (iii) Membrane adsorption can be controlled by modifying the order/disorder of the RNA sequence, for example, by chemical modifications that alter RNA rigidity. Overall, our study provides valuable information that can be used to expand the current applications of lipid–oligonucleotide conjugates.

## Supporting Information Available

Running average of the minimum distance between oligomers and DPPC-gel bilayer, comparison of different oligomers’ density profiles, probability distribution of distances between the COMs of oligomers and bilayer headgroup, interaction energy (*E*_Coul._ + *E*_LJ_) per nucleotide between the various short oligomers and the DPPC-gel bilayer, representative snapshot from MD simulation of A4 oligomer/DPPC-gel bilayer system in the presence of ions, minimum distance between dsRNA, G-rich ssRNA, A24, U24, and DPPC-gel bilayer, radius of gyration of ssRNAs over the simulation time, interaction energy per nucleotide ((*E*_Coul._ + *E*_LJ_) over time for phosphates (*P*O_4_^–^) of dsRNA or U24 backbone with DPPC-gel bilayer and all atoms of dsRNA or U24 with DPPC-gel bilayer, DPPC-RNA H-bonds in dsRNA or ssRNA/DPPC-gel bilayer systems, comparison analysis of minimum distance between ssRNA and DPPC-gel bilayer and radius of gyration of ssRNA in the absence and presence of physiological salt, molecular structures of nucleic bases, MD simulation analysis of oligomers–DPPC bilayer H-bonds, details about the systems simulated and RNA sequences used in the study (PDF)
