# Supplementary material for: RNA Order Regulates Its Interactions with Zwitterionic Lipid Bilayers
Source: Nano Lett. 2024 Dec 24;25(1):77–83. doi: 10.1021/acs.nanolett.4c04153 (PMC11719626; doi:10.1021/acs.nanolett.4c04153)
Supplement: Supplementary file 1 — nl4c04153_si_001.pdf [file nl4c04153_si_001.pdf]

Supporting Information for

RNA order regulates its interactions with zwitterionic lipid bilayers

Akhil Pratap Singh[1], Janak Prabhu[1] and Stefano Vanni[1,2]*

1. Department of Biology, University of Fribourg, Chemin du Musée 10, Switzerland
2. Swiss National Center for Competence in Research (NCCR) Bio-inspired Materials, University of Fribourg, Chemin des Verdiers 4, CH-1700 Fribourg, Switzerland

*Email- stefano.vanni@unifr.ch

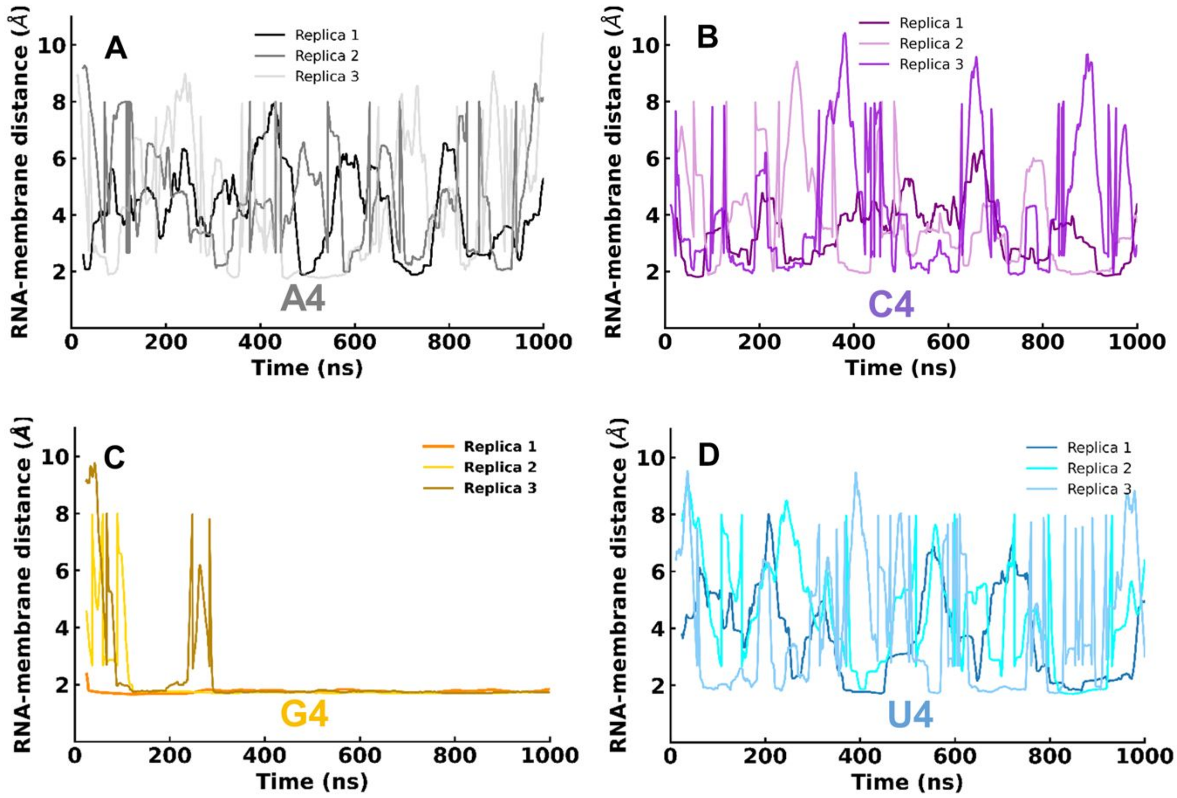

Figure S1. Running average of the minimum distance between oligomers and the DPPC-gel bilayer over simulation time.

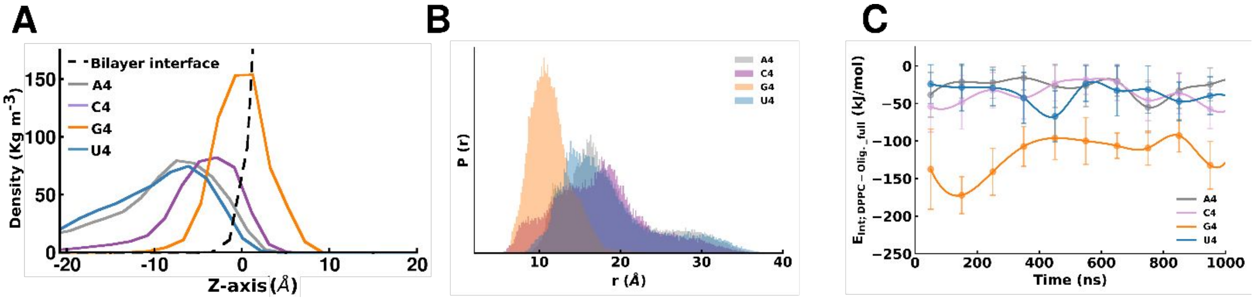

Figure S2. ***(A)*** Comparison of different oligomers density profiles along the normal of the DPPC bilayer plane. ***(B)*** Probability distribution of distances between the center of mass (COMs) of oligomers and bilayer head group observed in the complete trajectories (1μs). (***C***) Interaction energy ($E_{\text{Coul.}} + E_{LJ}$) per nucleotide between the various oligomers and the DPPC-gel bilayer over time. Error bars on the energy curves were estimated using block averaging.

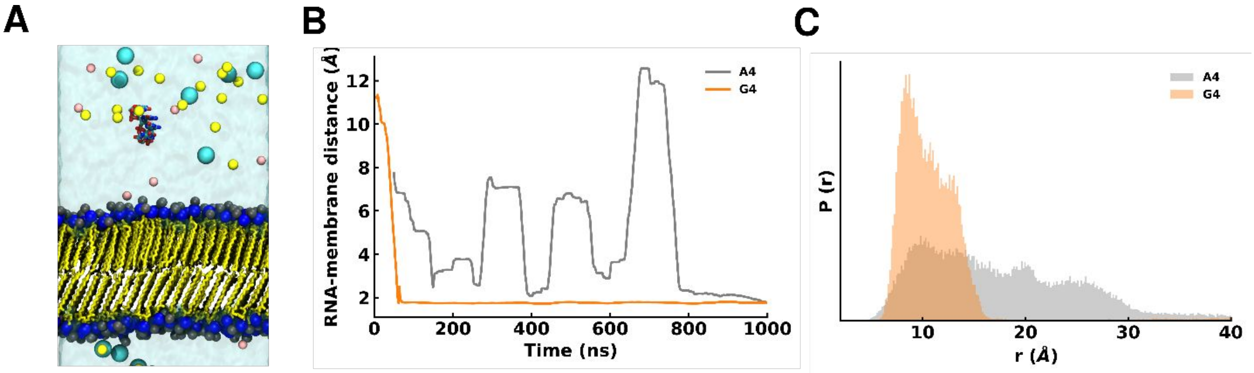

Figure S3. *(A)* Representative snapshot from MD simulation of A4 oligomer/DPPC-gel bilayer system in presence of *150mM NaCl* and *50 mM $MgCl_2$* ions. Color scheme: Blue and black for nitrogen and phosphate atom of lipid head groups, yellow for lipid tails, translucent ice blue for water, light yellow spheres for sodium ions, cyan for chloride ions, pink for magnesium ions. The A4 oligomer is in multicolored licorice representation. *(B)* Running average of the minimum distance between oligomers and lipid bilayer in A4/DPPC-gel bilayer and G4/DPPC-gel bilayer systems, and *(C)* probability distribution of distances between the center of mass (COMs) of oligomers and bilayer head group observed in complete trajectory.

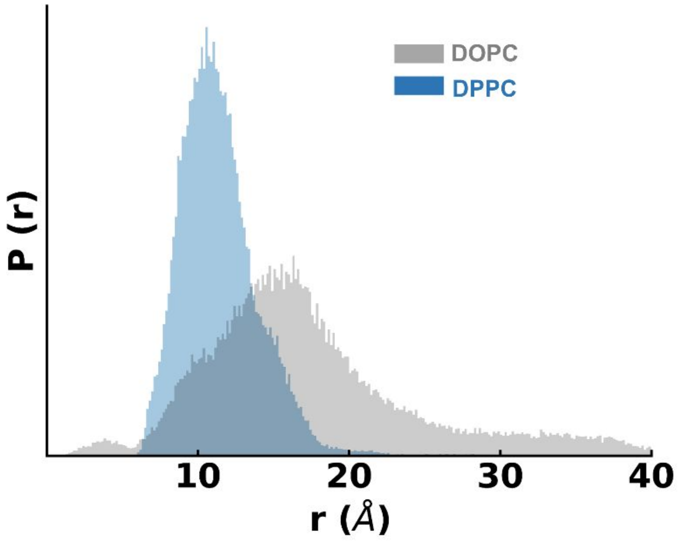

Figure S4. Probability distribution of distances between the center of mass (COMs) of the G4 oligomer and DPPC-gel (blue) and DOPC (gray) bilayer head group observed in the MD simulations.

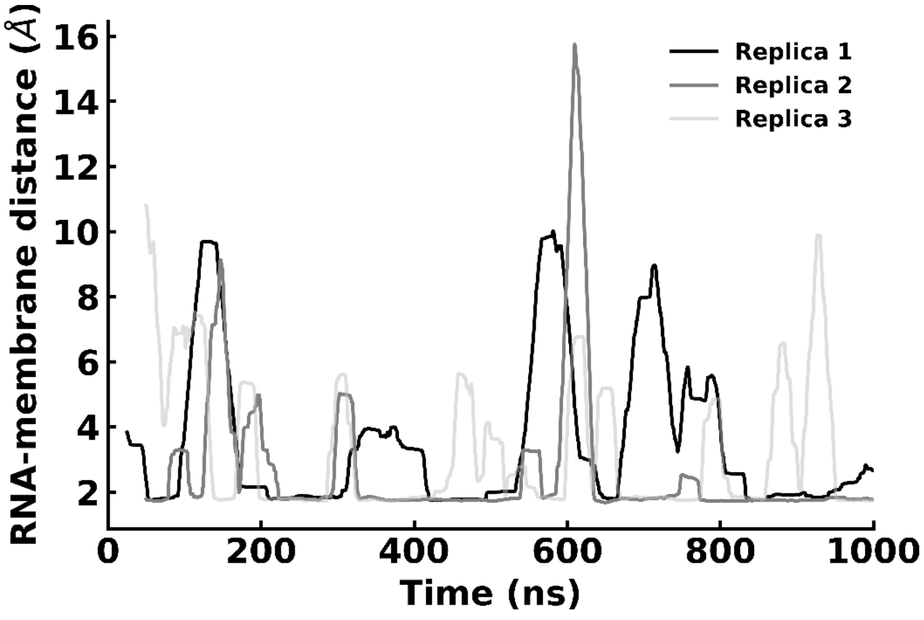

Figure S5. Running average of the minimum distance between G4 and the DOPC bilayer over simulation time.

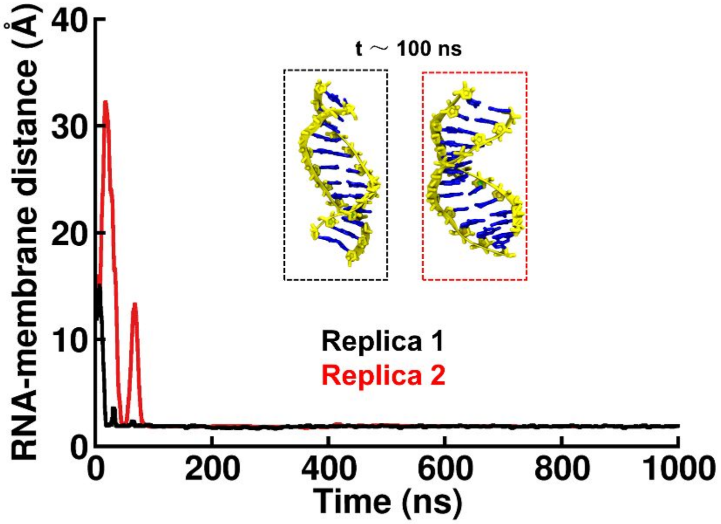

Figure S6. Minimum distance between dsRNA and DPPC-gel bilayer. Inset: structural arrangement of dsRNA in both dsRNA/ DPPC-gel bilayer replicas at t = 100 ns. Both replicas show quick absorption of dsRNA at DPPC-gel bilayer.

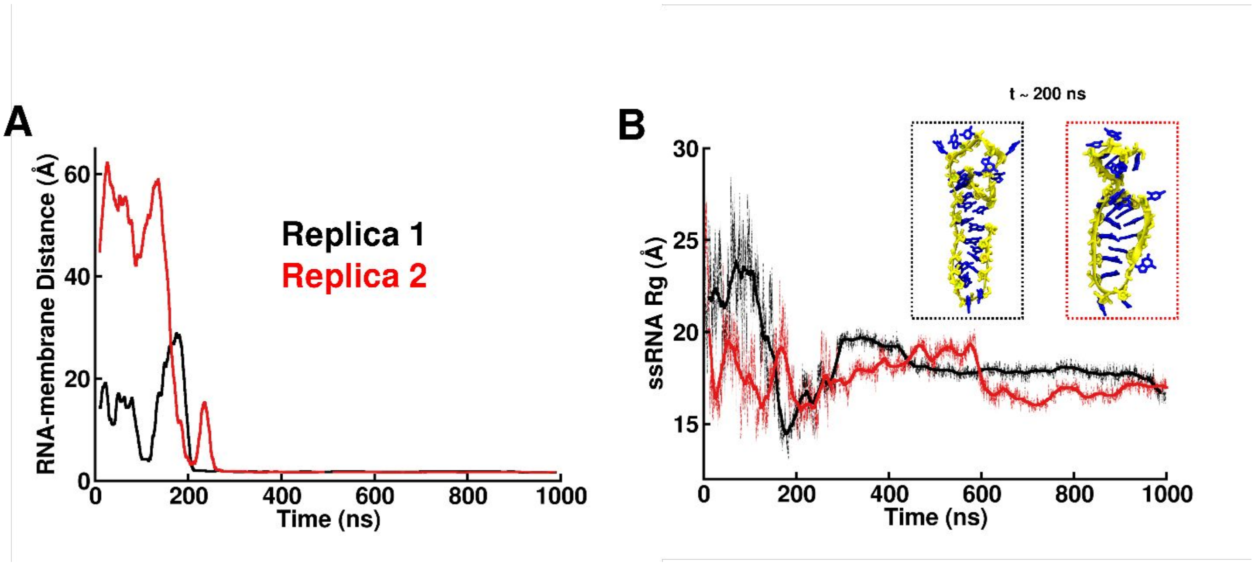

Figure S7. (A) Minimum distance between G-rich ssRNA and DPPC-gel bilayer. (B) Radius of gyration G-rich ssRNA over the simulation time. Inset: structural arrangement of G-rich ssRNA in both ssRNA/DPPC-gel bilayer replicas at t = 300 ns. Fig S5A and Fig S5B show that ssRNA took approximately 200 ns to fold, which concomitantly led to adsorption to the DPPC-gel bilayer.

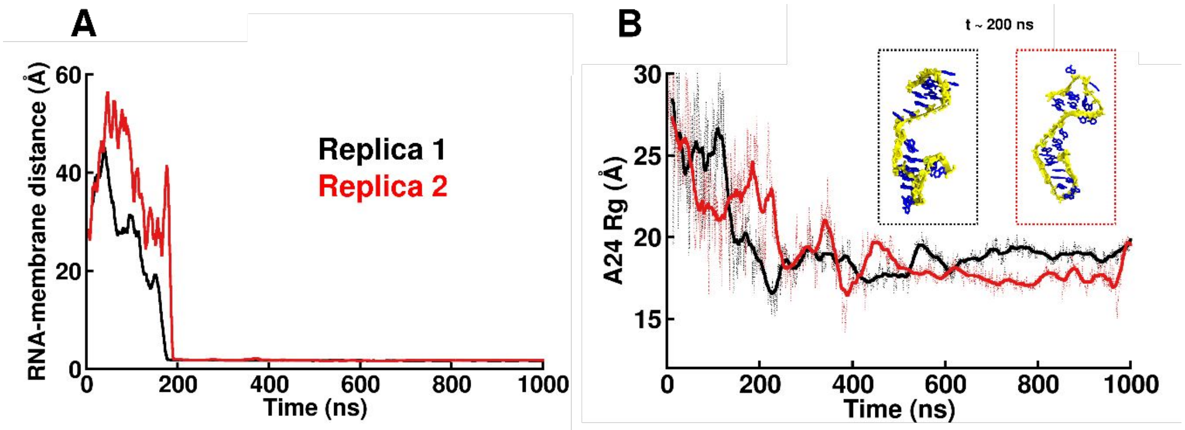

Figure S8. *(A)* Minimum distance between A24 and DPPC-gel bilayer. *(B)* Radius of gyration of A24 over the simulation time. Inset: structural arrangement of *A24* in both A24/DPPC-gel bilayer replicas at t = 200 ns. Fig S6A and Fig S6B show that *A24* took approximately 200 ns to fold through base-base stacking, which concomitantly led to adsorption to the DPPC-gel bilayer.

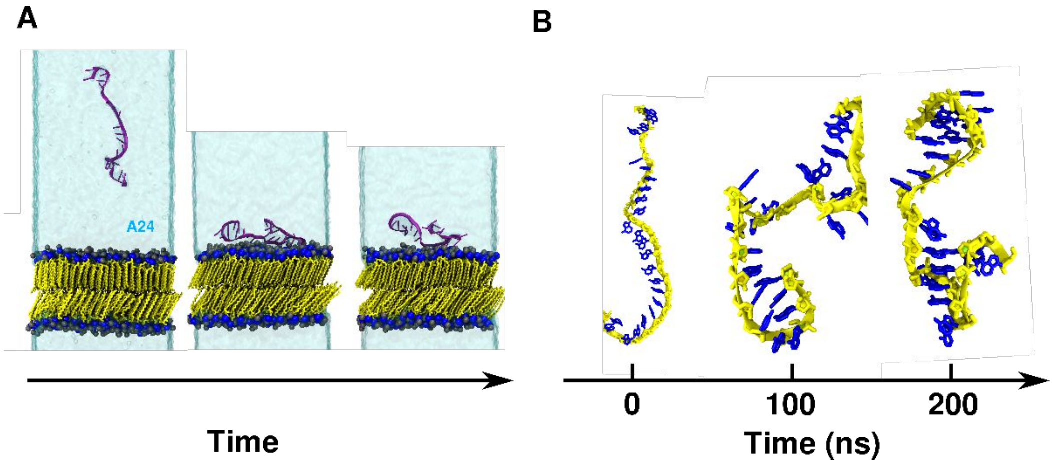

Figure S9. (A) Representative snapshots from MD simulations of the A24/DPPC-gel bilayer system. Color scheme: Blue and black for nitrogen and phosphate atom of lipid head groups, yellow for lipid tails, translucent ice blue for water. RNA structures are depicted in new-cartoon (magenta) representation. *(B)* Structural rearrangement of A24 in the A24/DPPC-gel bilayer system over time. Color scheme: yellow for A24 backbone and blue for nucleic bases.

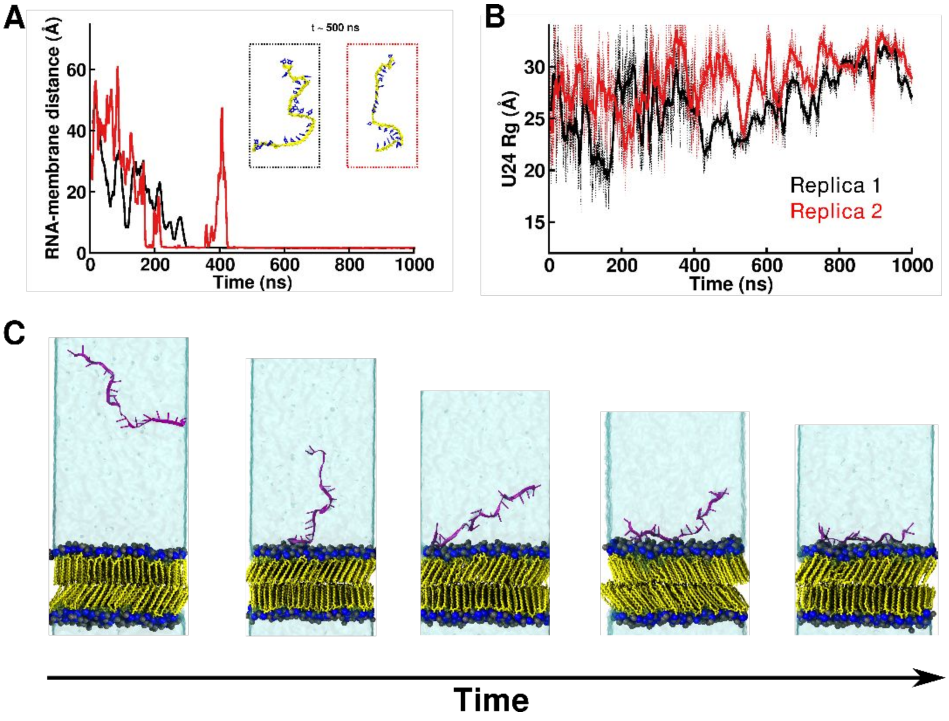

Figure S10. *(A)* Minimum distance between U24 and DPPC bilayer. Inset: Structural arrangement of *U24* in both U24/DPPC-gel bilayer replicas after adsorption (at t = 500 ns). *(B)* Radius of gyration of U24 over the simulation time. *(C)* Representative snapshots from MD simulations of the U24/DPPC-gel bilayer systems. Color scheme same as the Fig. S2.

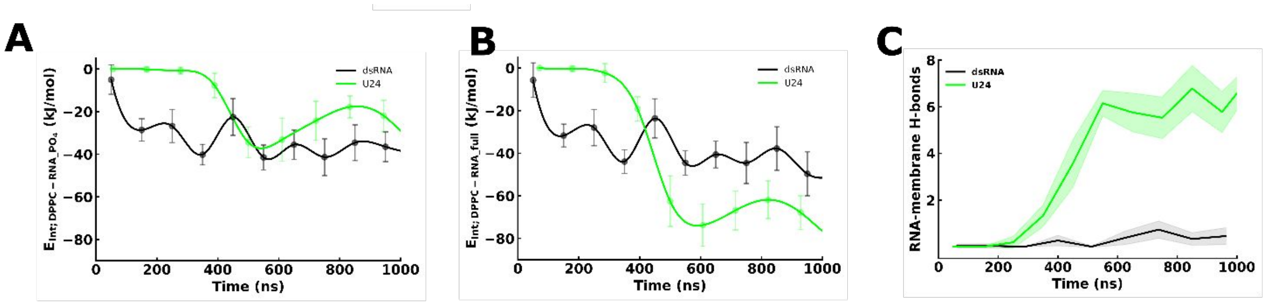

Figure S11. Interaction energy per nucleotide $(E_{\text{Coul.}} + E_{LJ})$ over time. *(A)* phosphates (PO_4^-) of dsRNA or U24 backbone with DPPC-gel bilayer, *(B)* All atoms of dsRNA or U24 with DPPC-gel bilayer. (C) DPPC-RNA H-bonds in dsRNA or ssRNA/DPPC-gel bilayer systems, as a function of time. Solid line indicates the running average; shade area indicates the standard deviation.

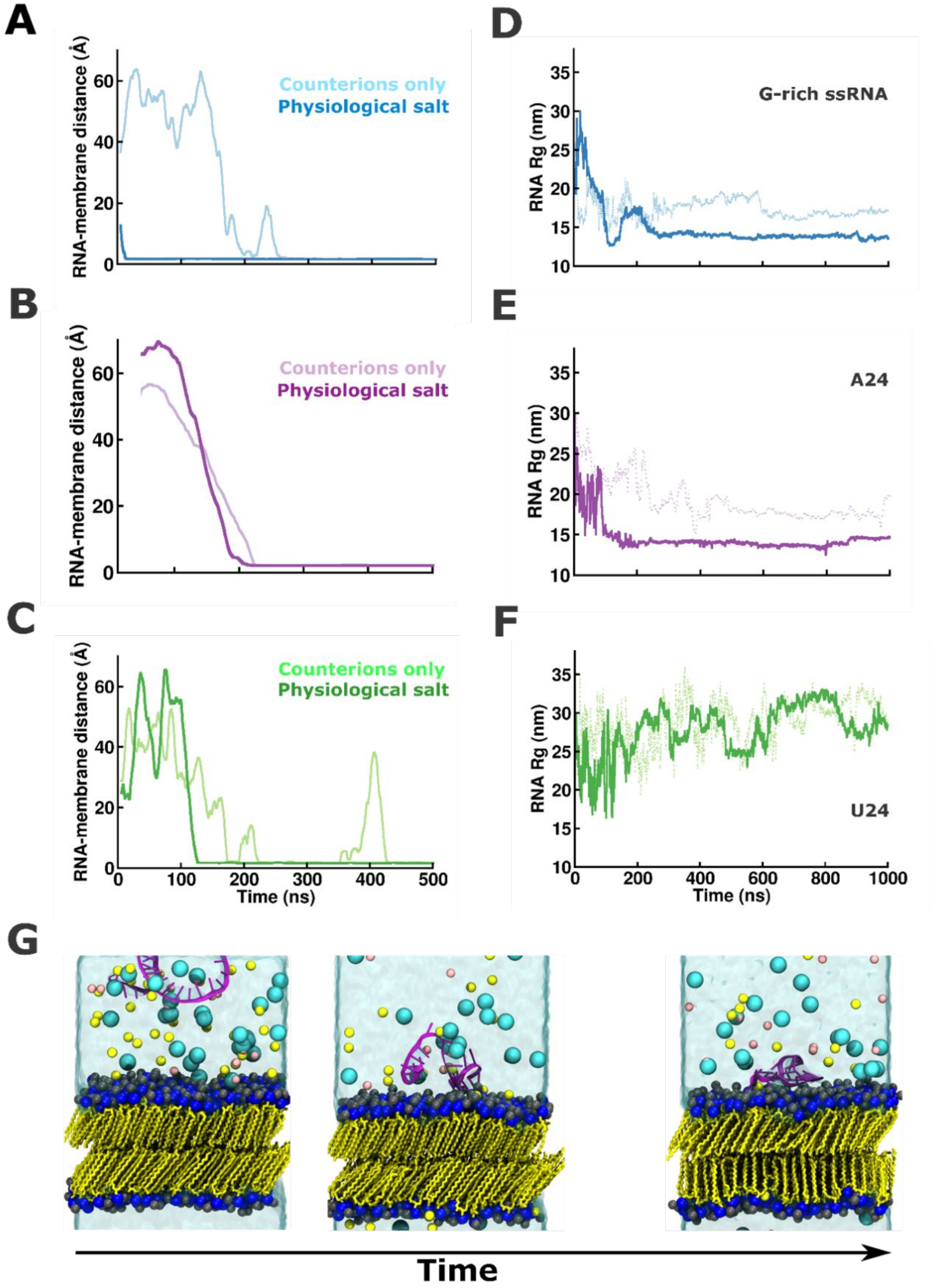

Figure S12. Comparison analysis of minimum distance between ssRNA and DPPC-gel bilayer *(A, B, C)*, and *corresponding* radius of gyration of ssRNA *(D, E, F)* in absence and presence of physiological salt (150mM NaCl + 50 mM $MgCl_2$). *(C)* Representative snapshots from MD simulations of G-rich ssRNA/DPPC-gel bilayer systems in presence of physiological salt. Color scheme same as the Fig. S2A.

(A)

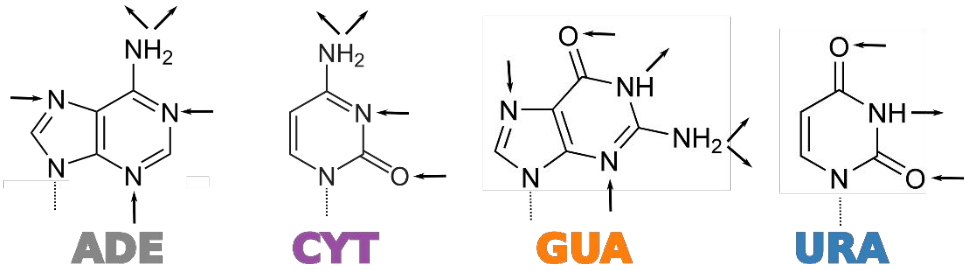

(B)

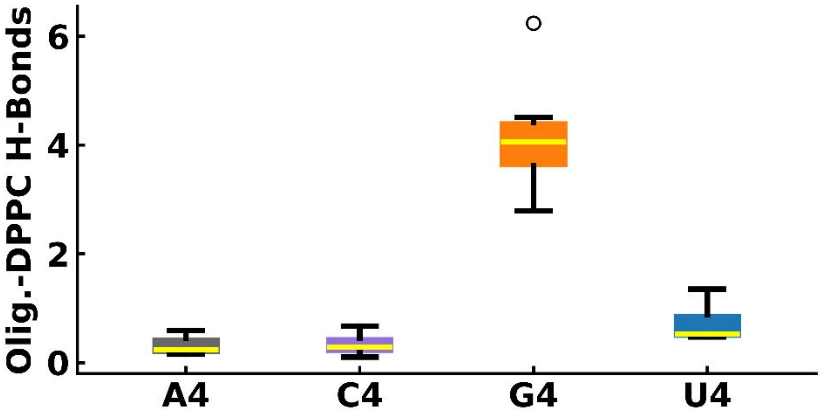

Figure S13. (A) Molecular structures of nucleic bases, showing the number of interaction sites (acceptor and donor atoms). Guanine has higher acceptor and donor H-atoms to form H-bonds. **(**B) MD simulation analysis of oligomers-DPPC bilayer H-bonds. Color scheme: gray for A4, purple for C4, orange for G4 and blue for U4 oligomers.

Table S1. Details about the systems simulated in this study.

System name	Lipids per Leaflet/Total	Length of water slab in Z-direction (Å)	Number of replicates
A4/DPPC-gel bilayer	64/128	80	3
C4/DPPC-gel bilayer	64/128	80	3
G4/DPPC-gel bilayer	64/128	80	3
U4/DPPC-gel bilayer	64/128	80	3
G4/DOPC-liquid bilayer	64/128	80	3
dsRNA/DPPC-gel bilayer	144/288	180	2
dsRNA/DOPC-liquid bilayer	144/288	180	2
G-rich-ssRNA/DPPC-gel bilayer	144/288	180	2
A24-ssRNA/DPPC-gel bilayer	144/288	180	2
U24-ssRNA/DPPC-gel bilayer	144/288	180	2

All systems are neutralized by just Na^{+} and Cl^{-} ions or an explicit physiological salt concentration of 150 mM $NaCl$ + 50 mM $MgCl_2$.

Table S2. Details about RNA sequences used in the study.

Sequence name	Sequence
A4	AAAA
C4	CCCC
G4	GGGG
U4	UUUU
4JRT	GGGUGGUGCGGG<br>CCUGCACUGCCC
G-rich ssRNA	GUGGUGCGGGGGGGUGGUGCCGG
A24 ssRNA	AAAAAAAAAAAAAAAAAA AAAAAA
U24 ssRNA	UUUUUUUUUUUUUUUUUUUUUUUU

Supplementary Methods

Simulations Details. All MD simulations in this work were carried out with the simulation software GROMACS, version 2021.5.[1-3] DPPC and DOPC lipid bilayer structures were built using the CHARMM-GUI package, [4-5] and then equilibrated for 50 ns at 298K and 310K, respectively, and 1 atm. The resulting configurations were used as the initial structure to build the final model systems: lipid bilayers in the presence of short-chain oligomers, either double-stranded RNA (dsRNA) or single-stranded RNA (ssRNA), through PACKMOL.[6] Ions were placed randomly in the simulation box. Short-chain oligomers and ssRNA PDB structures were determined using the RNAComposer software.[7] The dsRNA structure of 4JRT[8] was taken from the Protein Data Bank (PDB). The *CHARMM36* forcefield was employed for all simulations.[9-12] CHARMM TIP3P water model[13,14] was used to solvate all systems. TopoTools, the virtual molecular dynamics (VMD) plugin, was used to convert PSFGEN generated topologies into GROMACS file formats.[15,16] All simulations were performed with a 2 fs time step, and data was saved every 10 ps. The temperature (298 K or 310 K) was maintained with the Nosé−Hoover thermostat,[17,18] and the semi-isotropic Parrinello−Rahman barostat was used to control the pressure.[19] All the nonbonded interactions were truncated at a cutoff distance of 12 Å. The Particle Mesh Ewald (PME) method was used for the efficient computation of long-range electrostatic using a real space cutoff of 12 Å. Periodic boundary conditions (PBC) were applied in all directions.

All systems were minimized using steepest descent algorithm to eliminate any possible clashes and bad contacts. Subsequently, an NVT ensemble simulation was conducted for 2 ns using position restraints to all heavy atoms. Then, NPT ensemble simulations were performed for 5 ns for further equilibration. Finally, production runs were carried out for 1μs for all systems at the above described conditions, with no restraints. The simulation time have been chosen based on prior MD simulations studies [20-24] , and to obtain convergence for multiple observables, including interaction energy (Figs S1, S9) and radius of gyration (Figs S5, S6, S8, S10). Selected snapshots were rendered using the VMD software package.

Binding free energy. The potential of mean force (PMF) for the A4, C4, G4, and U4 oligomers binding to the DPPC-gel bilayer as a function of distance was computed using the umbrella sampling algorithm[25] and the weighted histogram analysis method (WHAM).[26] The oligomers was

pulled from bound state to unbound state along the direction of the bilayer normal (z-axis) with a force constant of 1200 kJ mol^{-1} nm^{-2}, for a total of 35 windows with a window spacing of 0.1 nm. All windows were equilibrated for 1 ns and then used as starting configurations for umbrella sampling simulations. Each window was simulated for 300-500 ns. Error bars on the PMF curves are estimated using block averaging.

Interaction Energy. The interaction energy based on short-range energy components was calculated using GROMACS energy groups[27-31] by decomposing the potential energy *via* rerunning the trajectories as follows:

$$\langle E_{\text{interaction}} \rangle = \langle E_{\text{LJ}} \rangle + \langle E_{\text{Coulomb}} \rangle$$

Hydrogen bond definition. We analyzed hydrogen bonds (H-bonds) between all possible donors and acceptors atoms with the GROMACS program *gmx hbond*. It characterizes an H-bond between a "donor" atom (a hydrogen connected to oxygen or other electronegative atom) and "acceptor" atom (oxygen or another electro-negative atoms) by two parameters: the radial distance between electronegative atoms, r, and the angle formed between the hydroxyl and the vector connecting the oxygen atoms, θ. To determine if an H-bond exists, a geometrical criterion was used: r is $\leq$ 3.5 Å and θ $\leq$ 30°.[32-34]

Radius of gyration.

We calculated the radius of gyration (R_g) with the GROMACS program *gmx gyrate* as follows:

$$R_g = \sqrt{\frac{\sum_i \|r_i\|^2 m_i}{\sum_i m_i}}$$

where m_i and r_i are the mass and the position of atom *i* with respect to the center of mass of the molecule, respectively.

Cluster analysis and conformational entropy.

Oligomer conformational entropy is associated with the number of its attainable conformational microstates and respective probabilities as such:

$$S = -K_B \sum_{n=1}^{W} P_n ln\, P_n$$

where K_B is the Boltzmann constant, W is the number of unique conformational basins, and P_n is the probability of being in conformational basin *n*. The unique conformations were determined using cluster analyses on MD trajectories performed by the GROMACS program *gmx cluster*. We used *gromos* algorithm for clustering.[35] This method performs a clustering procedure based on the values of root-mean-squared deviation (RMSD). We choose the oligomer backbone for least squares fit and RMSD calculation with 0.15 nm cut-off value.

Relative shape anisotropy.

The relative shape anisotropy κ^2 is obtained from estimates of the radius of gyration R_g, asphericity *b*, and acylindricity *c* as follows:

$$\kappa^2 = \frac{b^2 + \left(\frac{3}{4}\right)c^2}{R_g^4}$$

where $R_g$is the radius of gyration, *b* the asphericity, and ***c*** the acylindricity: $R_g^2 = \lambda_x^2 + \lambda_y^2 + \lambda_z^2$, *b* = (3/2)$\lambda_z^2$ –(1/2) R_g^2, ***c*** = $\lambda_y^2 - \lambda_x^2$; $\lambda_x^2 \leq \lambda_y^2 \leq \lambda_z^2$, are the principle moments of the gyration tensor **S.**[36]

Supplementary References

(1) Abraham, M. J.; Murtola, T.; Schulz, R.; Páll, S.; Smith, J. C.; Hess, B.; Lindah, E. GROMACS: High Performance Molecular Simulations through Multi-Level Parallelism from

Laptops to Supercomputers. *SoftwareX* **2015**, *1–2*, 19–25. https://doi.org/10.1016/J.SOFTX.2015.06.001.

(2) Lindahl, E.; Hess, B.; van der Spoel, D. GROMACS 3.0: A Package for Molecular Simulation and Trajectory Analysis. *J Mol Model* **2001**, *7* (8), 306–317. https://doi.org/10.1007/S008940100045/METRICS.

(3) *GROMACS 2021.5 Source code*. *https://zenodo.org/records/5850051* (accessed 2024-05-25).

(4) Wu, E. L.; Cheng, X.; Jo, S.; Rui, H.; Song, K. C.; Dávila-Contreras, E. M.; Qi, Y.; Lee, J.; Monje-Galvan, V.; Venable, R. M.; Klauda, J. B.; Im, W. CHARMM-GUI Membrane Builder toward Realistic Biological Membrane Simulations. *J Comput Chem* **2014**, *35* (27), 1997–2004. https://doi.org/10.1002/JCC.23702.

(5) Jo, S.; Kim, T.; Iyer, V. G.; Im, W. CHARMM-GUI: A Web-Based Graphical User Interface for CHARMM. *J Comput Chem* **2008**, *29* (11), 1859–1865. https://doi.org/10.1002/JCC.20945.

(6) Martinez, L.; Andrade, R.; Birgin, E. G.; Martínez, J. M. PACKMOL: A Package for Building Initial Configurations for Molecular Dynamics Simulations. *J Comput Chem* **2009**, *30* (13), 2157–2164. https://doi.org/10.1002/JCC.21224.

(7) Sarzynska, J.; Popenda, M.; Antczak, M.; Szachniuk, M. RNA Tertiary Structure Prediction Using RNAComposer in CASP15. *Proteins: Structure, Function, and Bioinformatics* **2023**, *91* (12), 1790–1799. https://doi.org/10.1002/PROT.26578.

(8) Kondo, J.; Dock-Bregeon, A. C.; Willkomm, D. K.; Hartmann, R. K.; Westhof, E. Structure of an A-Form RNA Duplex Obtained by Degradation of 6S RNA in a Crystallization Droplet. *Acta Crystallogr Sect F Struct Biol Cryst Commun* **2013**, *69* (6), 634–639. https://doi.org/10.1107/S1744309113013018/TZ5032SUP1.PDF.

(9) Klauda, J. B.; Venable, R. M.; Freites, J. A.; O'Connor, J. W.; Tobias, D. J.; Mondragon-Ramirez, C.; Vorobyov, I.; MacKerell, A. D.; Pastor, R. W. Update of the CHARMM All-Atom Additive Force Field for Lipids: Validation on Six Lipid Types. *Journal of Physical Chemistry B* **2010**, *114* (23), 7830–7843. https://doi.org/10.1021/JP101759Q/SUPPL_FILE/JP101759Q_SI_001.PDF.

(10) Lee, J.; Cheng, X.; Swails, J. M.; Yeom, M. S.; Eastman, P. K.; Lemkul, J. A.; Wei, S.; Buckner, J.; Jeong, J. C.; Qi, Y.; et al. CHARMM-GUI Input Generator for NAMD, GROMACS, AMBER, OpenMM, and CHARMM/OpenMM Simulations Using the CHARMM36 Additive Force Field. *J. Chem. Theory Comput.* **2016**, *12* (1), 405–413. https://doi.org/10.1021/acs.jctc.5b00935.

(11) Denning, E. J. *et al.* Impact of 2'-Hydroxyl Sampling on the Conformational Properties of RNA: Update of the CHARMM All-Atom Additive Force Field for RNA. *J. Comput. Chem.* 2011, 32 (9), 1929-1943.
(12) Beglov, D.; Roux, B. Finite representation of an infinite bulk system: Solvent boundary potential for computer simulations *J. Chem. Phys.* **1994** 100, 9050–9063.
(13) Jorgensen, W. L.; Chandrasekhar, J.; Madura, J. D.; Impey, R. W.; Klein, M. L. Comparison of Simple Potential Functions for Simulating Liquid Water. J. Chem. Phys. 1983, 79, 926−935.
(14) Neria, E.; Fischer, S.; Karplus, M. Simulation of Activation Free Energies in Molecular Systems. *J Chem Phys* **1996**, *105* (5), 1902–1921. https://doi.org/10.1063/1.472061.
(15) Vermaas, J. V.; Hardy, D. J.; Stone, J. E.; Tajkhorshid, E.; Kohlmeyer, A. TopoGromacs: Automated Topology Conversion from CHARMM to GROMACS within VMD. *J Chem Inf Model* **2016**, *56* (6), 1112–1116.
(16) Humphrey, W.; Dalke, A.; Schulten, K. VMD: Visual Molecular Dynamics. *J Mol Graph* **1996**, *14* (1), 33–38. https://doi.org/10.1016/0263-7855(96)00018-5.
(17) Nosé, S. A Molecular Dynamics Method for Simulations in the Canonical Ensemble. *Mol Phys* **1984**, *52* (2), 255–268. https://doi.org/10.1080/00268978400101201.
(18) Hoover, W. G. Canonical Dynamics: Equilibrium Phase-Space Distributions. *Phys Rev A (Coll Park)* **1985**, *31* (3), 1695. https://doi.org/10.1103/PhysRevA.31.1695.
(19) Parrinello, M.; Rahman, A. Polymorphic Transitions in Single Crystals: A New Molecular Dynamics Method. *J Appl Phys* **1981**, *52* (12), 7182–7190. https://doi.org/10.1063/1.328693.
(20) Alavizargar, A.; Gass, M.; Krahn, M. P.; Heuer, A. Elucidating the Membrane Binding Process of a Disordered Protein: Dynamic Interplay of Anionic Lipids and the Polybasic Region. *ACS Phys. Chem. Au* **2024**, *4* (2), 167–179. https://doi.org/10.1021/acsphyschemau.3c00051.
(21) Ulmschneider, J. P.; Ulmschneider, M. B. Molecular Dynamics Simulations Are Redefining Our View of Peptides Interacting with Biological Membranes. *Acc. Chem. Res.* **2018**, *51* (5), 1106–1116. https://doi.org/10.1021/acs.accounts.7b00613.
(22) Herce, H. D.; Garcia, A. E. Molecular Dynamics Simulations Suggest a Mechanism for Translocation of the HIV-1 TAT Peptide across Lipid Membranes. *Proc. Natl. Acad. Sci. U. S. A.* **2007**, *104* (52), 20805–20810. https://doi.org/10.1073/pnas.0706574105.
(23) Rizo, J.; Sari, L.; Qi, Y.; Im, W.; Lin, M. M. All-Atom Molecular Dynamics Simulations of Synaptotagmin-SNARE-Complexin Complexes Bridging a Vesicle and a Flat Lipid Bilayer. *Elife* **2022**, *11*, 1–28. https://doi.org/10.7554/eLife.76356.

(24) Baylon, J. L.; Tajkhorshid, E. Capturing Spontaneous Membrane Insertion of the Influenza Virus Hemagglutinin Fusion Peptide. *J. Phys. Chem. B* **2015**, *119* (25), 7882–7893. https://doi.org/10.1021/acs.jpcb.5b02135.

(25) Torrie, G. M. & Valleau, J. P. Nonphysical sampling distributions in Monte Carlo free-energy estimation: Umbrella sampling. *J. Comput. Phys*. **1977** 23, 187–199.

(26) Hub, J. S., De Groot, B. L. & Van Der Spoel, D. g_whams-a free Weighted Histogram Analysis implementation including robust error and autocorrelation estimates. *J. Chem. Theory Comput.* **2010**, 6, 3713–3720.

(27) Carvalho Martins, L.; Cino, E. A.; Ferreira, R. S. PyAutoFEP: An Automated Free Energy Perturbation Workflow for GROMACS Integrating Enhanced Sampling Methods. *J. Chem. Theory Comput.* **2021**, *17* (7), 4262–4273. https://doi.org/10.1021/acs.jctc.1c00194.

(28) Sahihi, M.; Faraudo, J. Computer Simulation of the Interaction between SARS-CoV-2 Spike Protein and the Surface of Coinage Metals. *Langmuir* **2022**, *38* (48), 14673–14685. https://doi.org/10.1021/acs.langmuir.2c02120.

(29) Wennberg, C. L.; Murtola, T.; Páll, S.; Abraham, M. J.; Hess, B.; Lindahl, E. Direct-Space Corrections Enable Fast and Accurate Lorentz-Berthelot Combination Rule Lennard-Jones Lattice Summation. *J. Chem. Theory Comput.* **2015**, *11* (12), 5737–5746. https://doi.org/10.1021/acs.jctc.5b00726.

(30) Frausto-Parada, F.; Várgas-Rodríguez, I.; Mercado-Sánchez, I.; Bazán-Jiménez, A.; Díaz-Cervantes, E.; Sotelo-Figueroa, M. A.; García-Revilla, M. A. Grammatical Evolution-Based Design of SARS-CoV-2 Main Protease Inhibitors. *Phys. Chem. Chem. Phys.* **2022**, *24* (8), 5233–5245. https://doi.org/10.1039/d1cp04159b.

(31) Tang, H.; Zhao, Y.; Yang, X.; Liu, D.; Shan, S.; Cui, F.; Xing, B. Understanding the PH-Dependent Adsorption of Ionizable Compounds on Graphene Oxide Using Molecular Dynamics Simulations. *Environ. Sci. Nano* **2017**, *4* (10), 1935–1943. https://doi.org/10.1039/c7en00585g.

(32) Van Der Spoel, D.; Van Maaren, P. J.; Larsson, P.; Tîmneanu, N. Thermodynamics of Hydrogen Bonding in Hydrophilic and Hydrophobic Media. *Journal of Physical Chemistry B* **2006**, *110* (9), 4393–4398. https://doi.org/10.1021/JP0572535/ASSET/IMAGES/MEDIUM/JP0572535E00014.GIF.

(33) Luzar, A.; Chandler, D. Hydrogen-bond kinetics in liquid water. *Nature,* **1996,** *379*, 55–57 (1996).

(34) A. Luzar, Resolving the hydrogen bond dynamics conundrum. *J. Chem. Phys.* ***2000***, ***113*** 10663–10675.

(35) Daura, X.; Gademann, K.; Jaun, B.; Seebach, D.; Van Gunsteren, W. F.; Mark, A. E. Peptide Folding: When Simulation Meets Experiment. *Angewandte Chemie - International Edition* **1999**, *38* (1–2), 236–240. https://doi.org/10.1002/(sici)1521-3773(19990115)38:1/2<236::aid-anie236>3.0.co;2-m.
(36) Theodorou, D. N.; Suter, U. W. Shape of Unperturbed Linear Polymers: Polypropylene. *Macromolecules* **1985**, *18* (6), 1206–1214. https://doi.org/10.1021/MA00148A028/ASSET/MA00148A028.FP.PNG_V03.
